# Combined ligand-observe ^19^F and protein-observe ^15^N,^1^H-HSQC NMR suggests phenylalanine as the key Δ-somatostatin residue recognized by human protein disulfide isomerase

**DOI:** 10.1038/srep19518

**Published:** 2016-01-20

**Authors:** Kirsty L. Richards, Michelle L. Rowe, Paul B. Hudson, Richard A. Williamson, Mark J. Howard

**Affiliations:** 1 Protein Science Group, School of Biosciences, University of Kent, Canterbury, Kent CT2 7NJ, UK

## Abstract

Human protein disulphide isomerase (hPDI) is an endoplasmic reticulum (ER) based isomerase and folding chaperone. Molecular detail of ligand recognition and specificity of hPDI are poorly understood despite the importance of the hPDI for folding secreted proteins and its implication in diseases including cancer and lateral sclerosis. We report a detailed study of specificity, interaction and dissociation constants (K_d_) of the peptide-ligand Δ-somatostatin (AGSKNFFWKTFTSS) binding to hPDI using ^19^F ligand-observe and ^15^N,^1^H-HSQC protein-observe NMR methods. Phe residues in Δ-somatostatin are hypothesised as important for recognition by hPDI therefore, step-wise peptide Phe-to-Ala changes were progressively introduced and shown to raise the K_d_ from 103 + 47 μM until the point where binding was abolished when all Phe residues were modified to Ala. The largest step-changes in K_d_ involved the F11A peptide modification which implies the C-terminus of Δ-somatostatin is a prime recognition region. Furthermore, this study also validated the combined use of ^19^F ligand-observe and complimentary ^15^N,^1^H-HSQC titrations to monitor interactions from the protein’s perspective. ^19^F ligand-observe NMR was ratified as mirroring ^15^N protein-observe but highlighted the advantage that ^19^F offers improved K_d_ precision due to higher spectrum resolution and greater chemical environment sensitivity.

Protein disulphide isomerase (PDI) is an abundant protein found within the lumen of the ER of all eukaryotes at concentrations estimated to be ~0.8% of total cellular protein^1^. It is capable of catalysing the correct folding of secreted proteins by acting as a catalyst for the oxidation, reduction and isomerisation of disulfide bonds, a major rate limiting step in the formation of many folded proteins. In addition to its catalytic redox activity, PDI also acts as a molecular chaperone and has been shown to bind a variety of polypeptides^2^.

Human Protein Disulfide Isomerase (hPDI) is a 57 kDa protein consisting of four thioredoxin-like domains^1^. Two of the domains, **a** and **a’**, are redox active and contain conserved cysteine residues in an active site motif of Cys-Gly-His-Cys. The other domains, **b** and **b’**, lack these conserved residues and are non-catalytic. Instead, the **b’** domain provides the principal binding site for ligand interaction^3^ and the **b** domain ensures alignment of the functional sites and may confer structural stability to the protein. There is also a short 19 amino acid **x-linker** region between the **b’** and **a’** domains allowing inter-domain flexibility, and a C-terminal acidic tail (**c**) with the ER retention signal KDEL. The complete domain architecture is **abb’xa’c**.

The ligand binding site on hPDI **b’x** has been mapped by NMR^4^,^5^,^6^. The domain contains a large multivalent binding pocket that spans the domain and is lined with solvent exposed hydrophobic side chains. The **b’** domain is capable of binding small peptides (10–15 amino acid residues) via hydrophobic interactions, independent of disulfide bonds or cysteine residues^2^,^7^. The **b’** domain, along with binding contributions from the redox active domains **a** and **a’**, is also essential for the binding of larger peptides and non-native proteins^8^,^9^,^10^. Having a large low-affinity binding site, displaying micromolar K_d_ values, allows hPDI to bind a wide range of protein folding intermediates and then release the correctly folded protein once the correct conformation is achieved^11^. However, micromolar affinities can be considered as relatively ‘weak’ binding events and create a challenge to measuring precise and meaningful dissociation constant data.

Obtaining detailed binding information is of considerable importance; ligand specificity is still poorly understood and as yet no structural models exist for hPDI bound to a target ligand. By unravelling the molecular nature of the hPDI-ligand interaction we hope to expand our understanding of the structure-function based specificity of hPDI. Furthermore, medical interest in hPDI and related family members has significantly increased as this protein family have been associated with several diseases^12^ including cancer^13^ and lateral sclerosis^14^. The only structural information on a PDI-ligand complex to date stems from the recently published thermophillic fungus (*Humicola insolens*) **b’xa’** PDI bound to a α-synuclein peptide (αSN) that identified this PDI as being able to capture a hydrophobic ligand segment with key contacts from valine and leucine αSN residues^10^. However, this binding site identified in *Humicola insolens* **b’** is located toward the N-terminal region of **b’** whereas the binding site in hPDI **b’** appears much larger and registers across the beta-sheet and residues in the C-terminal half of the protein including the **x-**linker^5^,^6^,^15^.

A well-documented method of measuring ligand binding by NMR is via chemical shift perturbation to calculate dissociation constants (K_d_)^16^,^17^. This is accomplished by collecting a set of spectra of the protein with varying concentrations of ligand. The overlaid spectra inform on the binding event and highlight the residues involved. ^19^F NMR has re-emerged as an important and valuable method for studying proteins and biomolecules as illustrated by several recent publications^15^,^18^,^19^,^20^,^21^,^22^,^23^,^24^,^25^. We report the combined use of backbone ^15^N/^1^H and ^19^F NMR chemical shift perturbations to study the molecular detail and specificity of the ligand binding mechanism of the hPDI fragment **b’x** with a known peptide ligand, Δ-somatostatin (Δ-som; AGSKNFFWKTFTSS)^2^,^26^. Interestingly, Δ-som has been extensively studied as a ligand for hPDI but it does not contain valine or leucine residues that provide key contacts for the αSN peptide to the thermophilic fungus PDI. We hypothesise that hPDI, as a protein-folding chaperone, can recognise a variety of hydrophobic amino acids and the key candidates in the peptide ligand Δ-som are phenylalanine and tryptophan. The combination of uniformly ^15^N enriched protein, selective peptide fluorination and Ala-substitutions in the peptide sequence has enabled a powerful NMR approach to monitoring ligand binding from both molecular perspectives (protein and ligand) that also highlights the potential importance of the phenylalanine over tryptophan for Δ-som recognition by hPDI.

## Results

**Figure 1:**
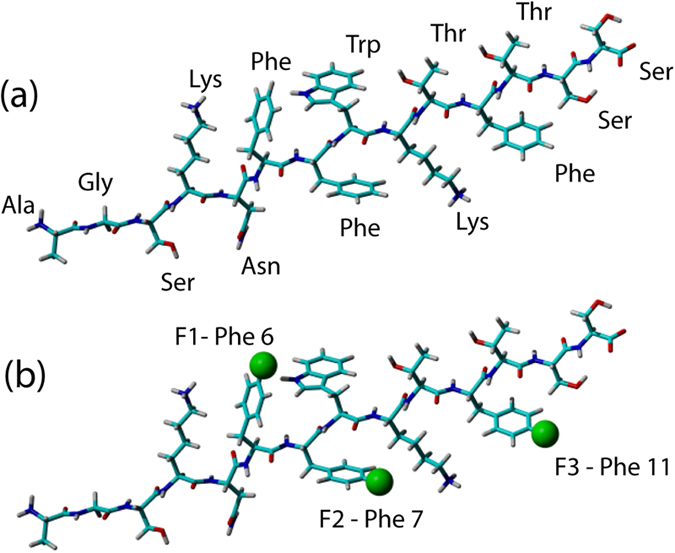
Extended stick models of Δ-somatostatin with (**a**) all amino acids labelled and (**b**) showing fluorinated phenylalanine residues together with nomenclature used throughout this study. Fluorine is shown as a green sphere in the 4-fluorophenylalaine positions in (**b**).

Figure 1 has been included to illustrate the nomenclature used to describe individual fluorinated Δ-som phenylalanine residues.

### Binding of Δ-somatostatin to hPDI b’x

Titration of Δ-som peptide into samples of **b’x** protein resulted in HSQC spectra with some 40 observable, assigned shifting amide peaks. Residues that exhibited significant chemical shift changes upon addition of peptide included Y310, L338 and W347 and have been previously identified as being involved in both peptide and larger ligand binding to hPDI **b’x** and **bb’x** constructs^5^,^6^,^11^. These residues map to the ligand-binding site or **x-linker** region that occupies the ligand binding site the **b’x** crystal structure^4^,^5^,^6^.

An average dissociation constant was calculated from the 40 individual residue fits giving a value of 103 ± 47 μM at 37 °C that is in good agreement with other studies that were more concisely investigated^5^,^6^,^15^,^27^. Supplementary Information Figure SIII contains example K_d_ data curve fits and demonstrate that despite the quality of fits, ^15^N-based K_d_ determination with ‘weak’ binding is prone to errors. Theoretically, this could be improved by titrating further to identify the curve plateau but is practically impossible do to limits with ligand solubility.

### Effect of fluorination of Δ-somatostatin binding to b’x

**Figure 2:**
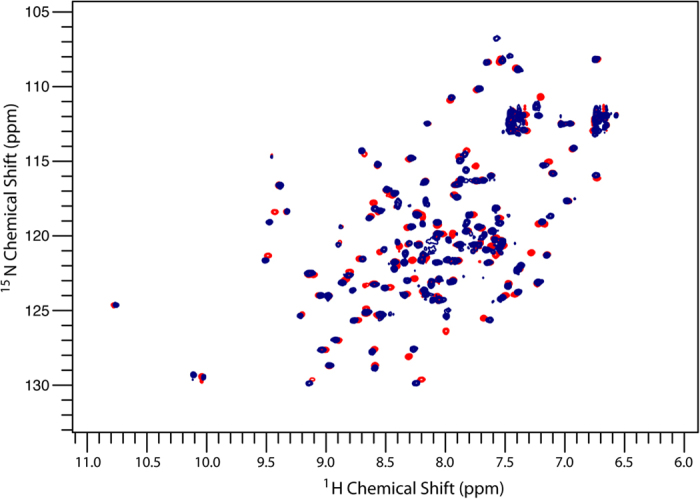
^15^N,^1^H- HSQC spectrum of 0.25 mM ^15^N b’x (blue) overlaid with 0.25 mM ^15^N b’x + 0.625 mM F1,2,3 Δ-som (red). Data were acquired for each spectrum (2048 × 256 points) over 45 minutes.

**Figure 3:**
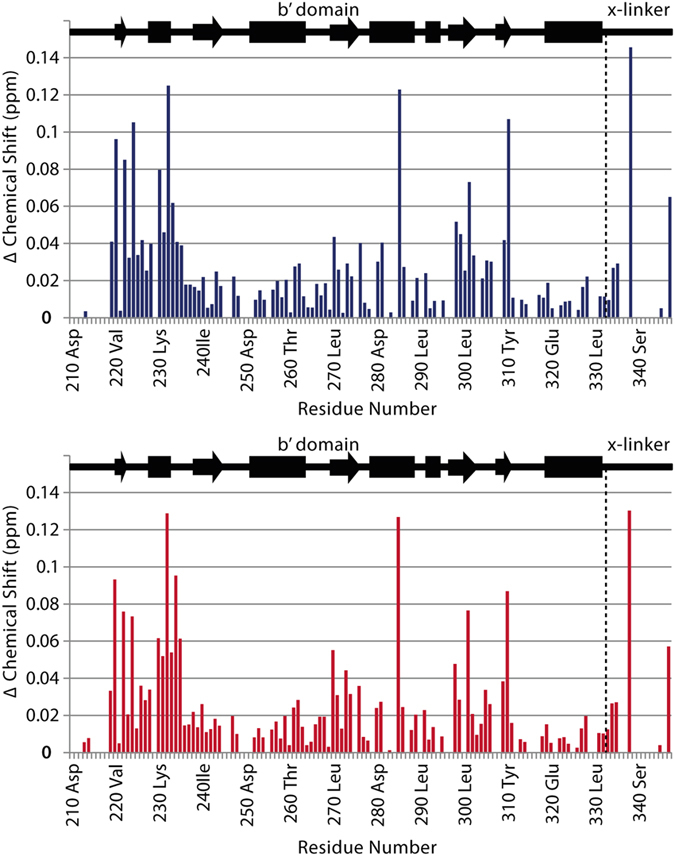
Chemical shift perturbation map of 0.25 mM ^15^N **b’x** and 0.625 mM Δ-som (blue) or 0.625 mM F1,2,3 Δ-som (red).

Analysis of ^15^N,^1^H-HSQC spectra of the protein in presence of triple-fluorinated F1,2,3 Δ-som shows the peptide binds identically to non-fluorinated peptide; resonances shift in the same direction and in similar magnitude (Fig. 2). Minimal chemical shift maps show that fluorinated and non-fluorinated peptide binding influence the same hPDI **b’x** residues and the magnitude of perturbation is similar for comparable peptide to protein ratios (Fig. 3 and Figure SIV).

Titration of the triple-fluorinated F1,2,3 Δ-som into 0.25 mM **b’x** protein and fitting the curve to equation (1) demonstrated that the peptide binds with an average K_d_ of 48 ± 35 μM, which whilst suggesting marginally tighter binding than the non-fluorinated peptide, is still within the error range. It was expected that fluorinated peptide would exhibit a lower K_d_ due to the increased hydrophobicity that fluorination contributes, strengthening hydrophobic interactions. The calculated K_d_ suggests a modestly tighter binding event with fluorinated peptide, but otherwise fluorinated Δ-som behaves similarly to the non-fluorinated peptide. It may be surprising that fluorinated aromatic residues exhibit increased hydrophobicity when fluorine is the most electronegative element but the phenomenon of increased hydrobicity for fluorinated amino acids has been reported before^28^,^29^.

Furthermore, fluorine is known to have a lipophilic effect when substituted into benzene compounds and log P values for fluorinated benzenes are higher than non-fluorinated benzenes.

### Ligand binding from the peptide’s view

Using single and triple fluorinated peptides provided a valuable insight into ligand binding from the peptide viewpoint. All three phenylalanine residues in Δ-som (Phe6, Phe7, Phe11) were fluorinated as it was anticipated they would be integral to the peptide’s binding interaction to hPDI.

**Figure 4:**
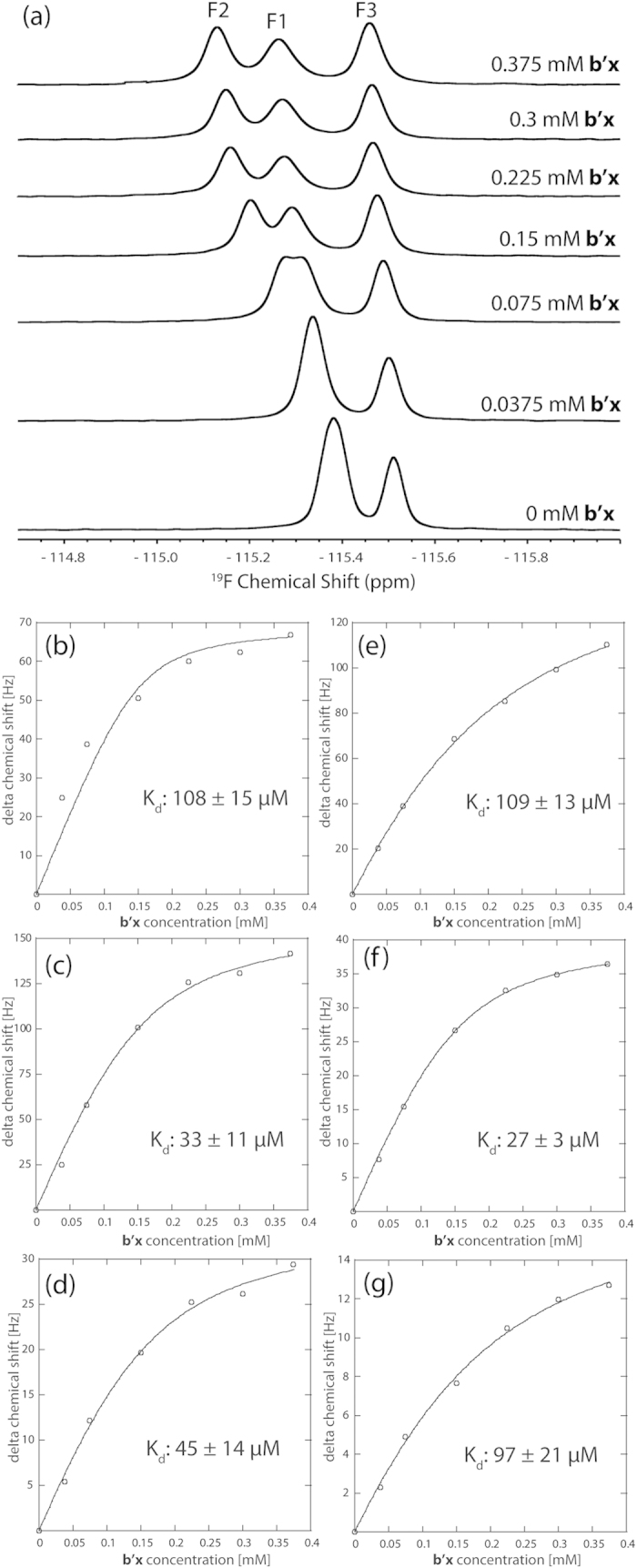
^19^F NMR spectra of 0.15 mM F1,2,3 Δ-som with increasing concentrations of ^15^N b’x protein (**a**). Titration curves of each fluorine resonance from the F1,2,3 Δ-som peptide: F1 peak (**b**), F2 peak (**c**), and F3 peak (**d**) upon addition of increasing ^15^N **b’x** protein. Fitting the points to equation (1) provides the curves and gives an average K_d_ of 62 + 13 μM. Titration curves of each fluorine resonance using singly-fluorinated F1 Δ-som (**e**), F2 Δ-som (**f**) and F3 Δ-som (**g**) upon addition of increasing ^15^N **b’x** protein are presented for comparison.

NMR titrations were carried out by collecting ^19^F NMR spectra (at a constant peptide concentration of 0.15 mM) and ^15^N,^1^H-HSQC spectra (at a constant protein concentration of 0.25 mM). This provided binding perspectives from both peptide and protein. ^19^F NMR data provided precise curve fits of equation (1) with K_d_ values in agreement with those obtained by ^15^N NMR (Table 1). The ^19^F data provides smaller curve fit errors, due to the simplified spectra, high signal to noise from the QCI-F cryoprobe and the significantly larger Hz/point resolution available from the ^19^F 1D experiment. Triple fluorinated peptide spectra are shown in Fig. 4 with example K_d_ graphs plotted using chemical shift change against **b’x** concentration (see Supplementary Information SV for single fluorinated peptide ^19^F spectra).

Dissociation constants from each ^19^F resonance in the triple-fluorinated peptide correlate well with those obtained from each single fluorinated peptide (compare Tables 1 and 2). Only F3 (Phe 11) reported a marginally lower K_d_ when all three Phe residues were fluorinated, suggesting that the Phe11 position is more sensitive to fluorination across the peptide. However, this difference was minor and did not result in a significantly different overall binding affinity. The binding event is considered to be bimolecular and cooperative; therefore it is important not to over interpret these subtle differences in K_d_ reported by each fluorinated position in Δ-som. However, the F2 position (Phe7) in both single and triple fluorinated peptides provides the lowest K_d_, with F1 (Phe6) and F3 (Phe11) consistently higher, suggesting that from this data the F2 (Phe7) position may be pivotal to the Δ-som interaction with **b’x**.

### Alanine substitutions of phenylalanine display weaker affinities and abolished binding

**Figure 5:**
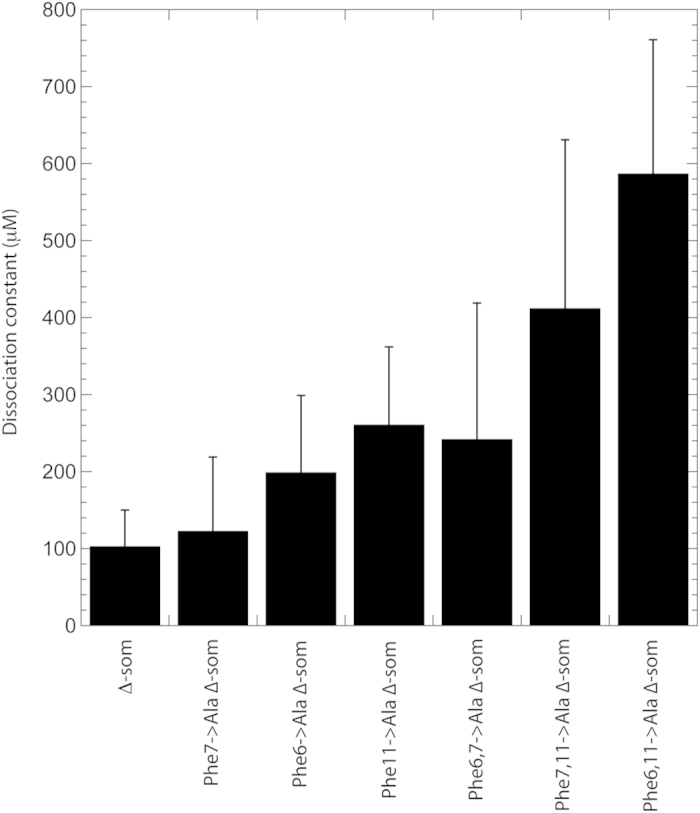
Histogram of dissociation constants (K_d_) of Δ-som and single and double alanine substituted Δ-som analogues obtained from ^15^N,^1^H- HSQC.

With the aim of revealing the importance of the Phe residues in Δ-som for the binding affinity to **b’x**, alanine substitutions were introduced at all phenylalanine positions. As a result three single Phe → Ala mutants, three double Phe → Ala mutants and one triple Phe → Ala peptide were synthesized and monitored for binding using ^15^N,^1^H-HSQC experiments that detected the protein. Results for these peptides are shown in Table 3 and displayed as a histogram in Fig. 5. All single Phe → Ala peptides exhibited weaker binding than Δ-som and whilst no single Phe residue was essential for binding, all three Phe residues do contribute to binding affinity. Data from the fluorinated peptides suggested that the F2 (Phe7) position may be pivotal, but the Phe7 → A change contradicts this as it provided least significant change in K_d_ by a single Ala mutant. This does suggest that fluorination influences the affinity, most likely due to the increased hydrophobicity it imparts on the peptide. Observing only single Phe → Ala changes suggests F3 (Phe11) substitution creates the largest increase in K_d_ and this trend was also observed in the double Ala mutants.

The double Ala mutants displayed weaker binding affinities than the single mutants and substitution of Phe11 to Ala has the largest increase in K_d_ that suggests the most significant disruption on the protein’s ability to recognise the peptide. The Phe6,7 → Ala substitution is less dramatic and provides a dissociation constant closest to single mutant peptide K_d_ values, whereas double substitutions involving Phe11 displayed weaker binding than Phe6,7 → Ala. The effect of Phe11 → Ala within both single and double mutations is is best demonstrated graphically in Fig. 5 and suggests an important role for Phe11 in Δ-som recognition by hPDI **b’x**. Phe11 was also shown to be highly variable in the ^19^F study; recall single and triple fluorinated peptides differences (see Fig. 4(d,g) and Tables 1 and 2). This sensitivity to fluorination at Phe11 could reflect its critical role in the peptide being recognised by hPDI. Acknowledging the role of Phe11 is interesting because it suggests that primary recognition of Δ-som involves the C-terminal region of the peptide. This is further supported by more insignificant changes in K_d_ between the single mutants and the Phe6,7 → Ala mutant. Figure 5 also highlights the issue of precision when measuring ‘weak’ micromolar binding. There is little doubt that as Δ-som is modified using progressive Phe → Ala changes, then K_d_ rises. However, Fig. 5 also demonstrates that the error involved also rises with K_d_ and demonstrates the practical issue of curve-fitting equation (1) to high micromolar K_d_ using a titration; high quality curve-fits require titration to approaching occupancy and plateau of the curve. In reality such fits difficult to achieve with high micromolar K_d_ systems, such as protein-folding chaperones like hPDI, because it requires a high concentration of ligand that is beyond the solubility limits of the system. High concentrations of biological molecules also provide additional sources of error such as aggregation and/or increases in viscosity. Although reducing the constant protein concentration can mitigate these issues, there is still a limit to detection to create sufficient signal to noise with adequate resolution to measure changes in chemical shift. The compromise was used here to provide the presented trends in increasing K_d_ with acceptable signal to noise, resolution and time efficiency of experiments. However, preliminary experiments provided comparable dissociation constants to those reported and provides confidence when inferring K_d_ trends discussed upon fluorination and alanine substitution.

The Phe6,7,11 → Ala Δ-som peptide did not provide any significant resonance shifts throughout the titration series and was classified as a non-binding peptide. More specifically, any shifts identified at the end point of the titration were extremely small (<0.01 ppm) and did not map to the ligand binding site (Supplementary Information Figure SVI). These extremely small shifts were randomly scattered on the protein’s surface and are similar to the distribution of shifts seen when using a non-binding control peptide; helix-28 from human serum albumin (RERQIKKQTALVELV) (Supplementary Information Figure SVII). The conclusion has to be that the phenylalanine residues in Δ-som are essential for recognition and binding by hPDI **b’x**.

## Discussion

This study presents a detailed analysis of peptide ligand binding to hPDI **b’x** using a combination of ^15^N/^1^H and ^19^F NMR spectroscopy. Mapping from both the peptide and the protein provides complementary information when measuring dissociation constants as well as the opportunity to interrogate residue specificity. A comparison of the **b’x** backbone chemical shift perturbations seen on binding of the fluorinated and non-fluorinated peptide confirms that the location of the binding site is unchanged and that the direction and extent of chemical shift perturbation is virtually identical in both cases suggesting a similar overall mode of interaction. Furthermore, chemical shift changes measured from fluorinated and non-fluorinated peptide-ligands are in very close agreement to previous studies and we have previously demonstrated that fluorination of hPDI **b’x** maintains the binding mode with Δ-somatostatin^15^. High-resolution 1D ^19^F NMR increased the precision of measuring K_d_ values when compared to ^15^N,^1^H backbone amide measurements from HSQC spectra and K_d_ values measured using fluorinated peptides were marginally smaller than the non-fluorinated counterpart which reflects a higher binding affinity. This observation of fluorination promoting hydrophobic interactions is in agreement with our previous work studying Δ-som binding to hPDI **b’x** using fluorinated protein that produced a K_d_ of 23 ± 4 μM at 25 °C^16^.

The alanine mutant Δ-som peptides demonstrate that all three phenylalanine residues contribute to the recognition of Δ-som by hPDI **b’x**. Removal of one or two of the phenylalanine residues did not abolish binding, although it did considerably weaken the affinity. However, removal of all three Phe residues from Δ-som prevented the peptide from interacting with **b’x** and our data suggests that the third phenylalanine residue, Phe11 (F3), makes the largest contribution towards binding affinity. This work highlights the importance of large, exposed, aromatic amino acids in binding to hPDI, much like other molecular chaperones^30^. However, it appears in the case of PDI, phenylalanine is primarily recognized in this example as a result of the following observations. The control peptide contains isoleucine, leucine and valine residues, with Leu and Val being implicated in the α-SN peptide as providing primary interactions with PDI **b’** from thermophilic fungus^10^. This may suggest *Humicola insolens* PDI has a distinctive mechanism of substrate recognition compared to human PDI. However, protein-folding chaperones are fluid and dynamic protein systems and it is possible that recognition is attributable to thermodynamics in addition to amino acids. All peptides used in this study were individually analysed by ^1^H NMR under the same buffer and temperature conditions used for the binding studies (data not shown). Peptide ^1^H NMR spectra were extremely similar and every spectrum displayed the same chemical shift limits with no dispersion. This is consistent with peptides in solution that have no apparent secondary structure or conformational differences. Therefore, fluorination or alanine substitution does not appear to facilitate any significant entropic changes and that the differences in dissociation constants are a result of direct interactions between ligand and target protein. This further supports our conclusions that Phe residues are important for recognition of Δ-som by hPDI and that changes are not due to induced thermodynamic difference by fluorination or alanine substitution.

As a final point regarding this study, we have also demonstrated a significant use for combined ^15^N and ^19^F labelling to probe ligand binding that also validates the role of ^19^F NMR to follow peptide binding from the ligand’s perspective. This approach enabled a hypothesis driven approach to demonstrating the importance of phenylalanine in Δ-somatostatin for substrate recognition by human PDI. This method can be applied to peptide-protein interaction studies where structure-function and molecular mechanistic knowledge is required. There are many different fluorinated amino acid options available within custom peptide synthesis that can be used as explicit probes that will subsequently inform a peptide mutagenesis strategy to test interaction hypotheses. This approach has the potential to be more specific and effective than alanine scanning, particularly with larger peptides. Furthermore, the ^19^F approach does not require uniform protein labelling, spectral assignment of the protein or costly isotopic peptide synthesis and such peptides are easily synthesized and cost little more than non-fluorinated peptides. In the example reported, both the peptide and protein inform on the binding event in a similar manner and fluorination of peptide residues did not significantly affect the peptide’s binding mode or affinity.

## Methods

### Recombinant Protein Expression and Purification

hPDI **b’x** was expressed in *E. coli* and purified as described previously^4^. Pure monomer species, isolated by gel filtration, was used for all NMR experiments (see Supplementary Information Figure SI for gel filtration trace).

### Peptide Ligands

Unlabelled Δ-som peptide (AGSKNFFWKTFTSS), 4-fluorophenylalanine (4-F) peptides: F1 (AGSKN(4-F)FWKTFTSS), F2 (AGSKNF(4-F)WKTFTSS), F3 (AGSKNFFWKT(4-F)TSS), F1,2,3 (AGSKN(4-F)(4-F)WKT(4-F)TSS), and Phenylalanine → Alanine (Phe → Ala) mutants: Phe6 → Ala (AGSKNAFWKTFTSS), Phe7 → Ala (AGSKNFAWKTFTSS), Phe11 → Ala (AGSKNFFWKTATSS), Phe6,7 → Ala (AGSKNAAWKTFTSS), Phe6,11 → Ala (AGSKNAFWKTATSS), Phe7,11 → Ala (AGSKNFAWKTATSS), Phe6,7,11 → Ala (AGSKNAAWKTATSS) and helix-28 from human serum albumin (RERQIKKQTALVELV) were generated by peptide synthesis and purified >95% by reverse-phase HPLC (Peptide Synthetics, Fareham). To solubilise the hydrophobic peptides, the lyophilised material was first dissolved in 100% D_6_-DMSO and then diluted 20-fold into the NMR sample. To test that DMSO did not influence the protein, ^15^N,^1^H-HSQC spectra were obtained with and without 5% DMSO and no changes were observed (data not shown).

### Nuclear Magnetic Resonance

All spectra were collected using a 4-channel, 5-amplifier Bruker Avance III 14.1 T (600 MHz ^1^H) NMR spectrometer equipped with a 5 mm QCI-F cryoprobe. ^15^N,^1^H-HSQC spectra were acquired over 45 min on samples containing 0.25 mM **b’x** and varying peptide concentrations. Each ^19^F 1D spectrum was acquired over 60 min on samples of 0.15 mM Δ-som with varying protein concentration. Ratios of the variable:static components in each experiment were 1:4, 1:2, 1:1, 1.5:1, 2:1 and 2.5:1. All samples were run at 310 K in 20 mM sodium phosphate buffer (pH 7.0) containing 50 mM NaCl, 5% D_6_-DMSO, 5% ^2^H_2_O and 0.05% sodium azide. 1D NMR data were processed using Bruker Topspin software and referenced using the position of trifluoroacetic acid (−76.55 ppm). 2D data were processed using NMRPipe^31^ and analysed using the CCPN Analysis software package^32^.

Amide chemical shifts were calculated in Hz as √[(0.6Δ^1^H)^2^ + (Δ^15^N)^2^]. Dissociation constants (K_d_) for each shifting peak were calculated by plotting the chemical shift perturbation (Hz) against ligand concentration (mM) and fitting the curve to the equation:


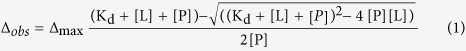


where Δ_obs_ is the observed chemical shift perturbation, Δ_max_ the maximum chemical shift perturbation and [P] and [L] the protein and ligand concentrations respectively. This equation was used to fit both ^15^N and ^19^F NMR data using KaleidaGraph 4.0 (Synergy Software). Standard errors from the Levenberg-Marquardt fitting routine were taken as the uncertainties. We determined K_d_ from ^15^N,^1^H HSQC NMR data using two approaches: First, by simultaneous fitting all shifts which are averaged to create a single plot, which is considered an accurate method of K_d_ determination^17^. Second, we tracked and fitted each peak individually to the equation above and averaged all results and errors across all fits. These analyses are summarised in Table S1 and demonstrates that both methods produce similar dissociation constants but the approach of simultaneous fitting created larger errors that were most like due to the relatively high K_d_ values determined. This hPDI system creates K_d_ values with shallow binding curve where errors where average chemical shifts reduce fitting accuracy and individual fitting produces higher resoltuion. Therefore, we used K_d_ values from the averaged individual fit method within this paper to report smaller errors. We believe this method works well for determining high K_d_ values because it encourages data to be triaged via inspection of individual curve fits and does not register discrepancies across the chemical shift or frequency fluctuation of binding.

### Backbone Assignment of b’x

The backbone assignment of the hPDI **b’x** construct at 25 °C was previously deposited in the BioMagResBank (accession code 15998)^5^. Temperature shift ^15^N,^1^H-HSQCs and HNCA and HN(CO)CA triple resonance experiments were carried out on ^15^N/^13^C labelled protein to assign the **b’x** backbone to 82% at 37 °C (see Supplementary Information Figure SII for assigned spectra).

## Additional Information

**How to cite this article**: Richards, K. L. *et al.* Combined ligand-observe ^19^F and protein-observe ^15^N,^1^H-HSQC NMR suggests phenylalanine as the key Δ-somatostatin residue recognized by human protein disulfide isomerase. *Sci. Rep.* **6**, 19518; doi: 10.1038/srep19518 (2016).

## Supplementary Material

Supplementary Information

## Figures and Tables

**Table 1:** Dissociation constants (K_d_) of Δ-som and single fluorinated Δ-som analogues obtained by ^15^N,^1^H-HSQC and direct ^19^F NMR titrations.

		PROTEIN ^15^N/^1^H Tracked/μM	LIGAND ^19^F Tracked (Single-F)/μM
AGSKNFFWKTFTSS	Δ-som	103 ± 47	N/A
AGSKNFFWKTFTSS	F1 Δ-som	61 ± 47	109 ± 13
AGSKNFFWKTFTSS	F2 Δ-som	51 ± 30	27 ± 3
AGSKNFFWKTFTSS	F3 Δ-som	84 ± 53	97 ± 21

Phe residue labelled for ^19^F NMR K_d_ determination is BOLD and underlined in the sequence. Standard errors from the Levenberg-Marquardt fitting routine of equation (1) were taken as the uncertainties.

**Table 2:** Dissociation constants (K_d_) of Δ-som and triple fluorinated Δ-som analogues obtained by ^15^N,^1^H-HSQC and direct ^19^F NMR titrations.

		PROTEIN ^15^N/^1^H Tracked/μM	LIGAND ^19^F Tracked (Triple-F)/μM
AGSKNFFWKTFTSS	Δ-som	103 ± 47	N/A
AGSKNFFWKTFTSS	F**1**,2,3 Δ-som	48 ± 35	108 ± 15
AGSKNFFWKTFTSS	F1,**2**,3 Δ-som	48 ± 35	33 ± 11
AGSKNFFWKTFTSS	F1,2,**3** Δ-som	48 ± 35	45 ± 14
AGSKNFFWKTFTSS	F**1,2,3** Δ-som	48 ± 35	62 ± 13*

Phe residue(s) used to measure K_d_ by ^19^F NMR are underlined in each sequence and shown in BOLD in column 2. Standard errors from the Levenberg-Marquardt fitting routine of equation (1) were taken as the uncertainties.

^*^F**1,2,3** Δ-som ^19^F tracked K_d_ is the mean K_d_ calculated from the result obtained from each monitored ^19^F resonance in the triple fluorinated peptide.

**Table 3:** Dissociation constants (K_d_) of single, double and triple alanine substituted Δ-som analogues calculated by ^15^N,^1^H- HSQC.

AGSKNFFWKTFTSS	^15^N Tracked/μM
Δsom	103 ± 47
Phe6 → Ala Δ-som	199 ± 100
Phe7 → Ala Δ-som	123 ± 96
Phe11 → Ala Δ-som	261 ± 101
Phe6,7 → Ala Δ-som	242 ± 177
Phe6,11 → Ala Δ-som	587 ± 174
Phe7,11 → Ala Δ-som	412 ± 219
Phe6,7,11 → Ala Δ-som	*Non binding*

Standard errors from the Levenberg-Marquardt fitting routine of equation (1) were taken as the uncertainties.
