# Supplementary material for: Combined ligand-observe 19F and protein-observe 15N,1H-HSQC NMR suggests phenylalanine as the key Δ-somatostatin residue recognized by human protein disulfide isomerase
Source: Sci Rep. 2016 Jan 20;6:19518. doi: 10.1038/srep19518 (PMC4726260; doi:10.1038/srep19518)

COMBINED LIGAND-OBSERVE ^{19}F AND PROTEIN-OBSERVE ^{15}N,^{1}H-HSQC NMR SUGGESTS PHENYLALANINE AS THE KEY Δ-SOMATOSTATIN RESIDUE RECOGNIZED BY HUMAN PROTEIN DISULFIDE ISOMERASE

KIRSTY L. RICHARDS, MICHELLE L. ROWE, PAUL B. HUDSON,
RICHARD A. WILLIAMSON* AND MARK J. HOWARD*

SUPPORTING INFORMATION

S. I: Gel filtration elution profile of ^{15}N labelled **b'x**

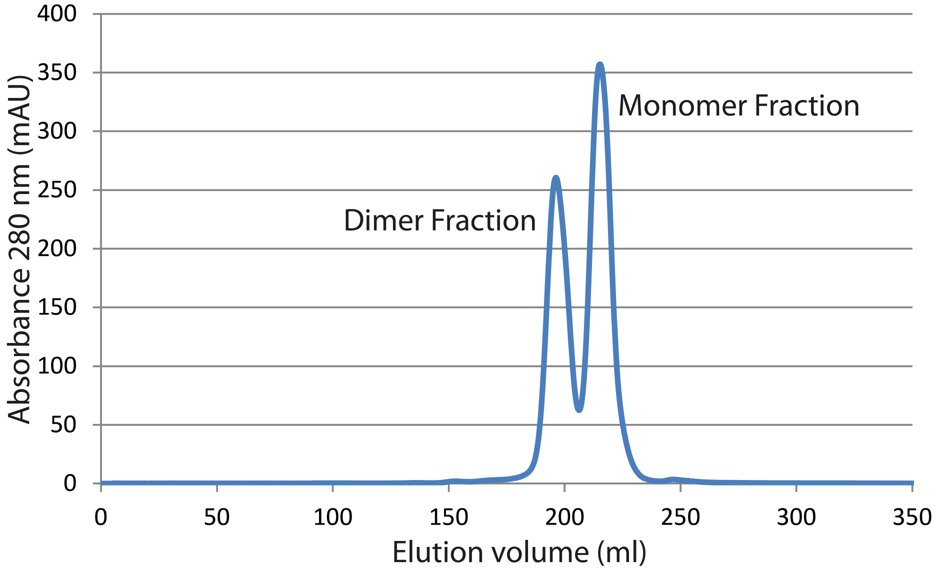

S. II: $^{15}N,^{1}H$-HSQC spectrum of 0.25mM ^{15}N **b'x** (blue) overlaid with 0.25mM ^{15}N **b'x** + 0.625mM Δ-som (red). Residue assignments for the **b'x** spectrum are shown.

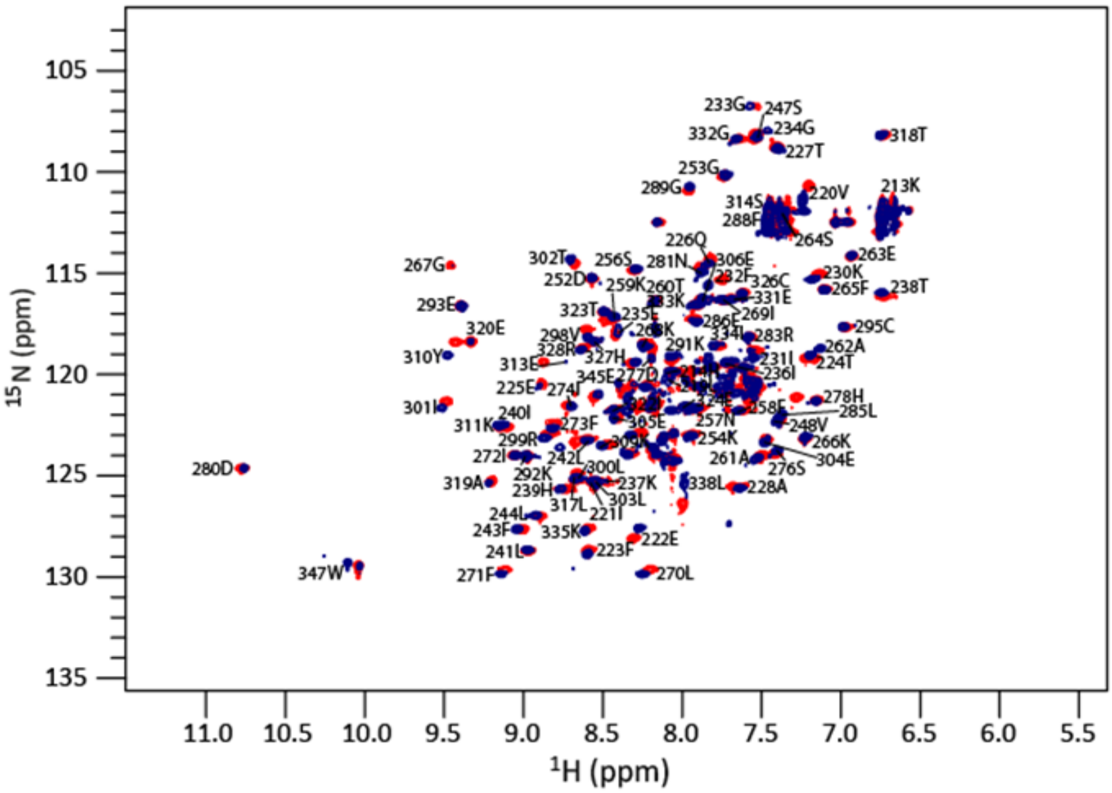

S. III: Example dissociation constant fits from $^{15}N,^{1}H$-HSQC data for 0.25mM ^{15}N **b'x** + Δ-som.

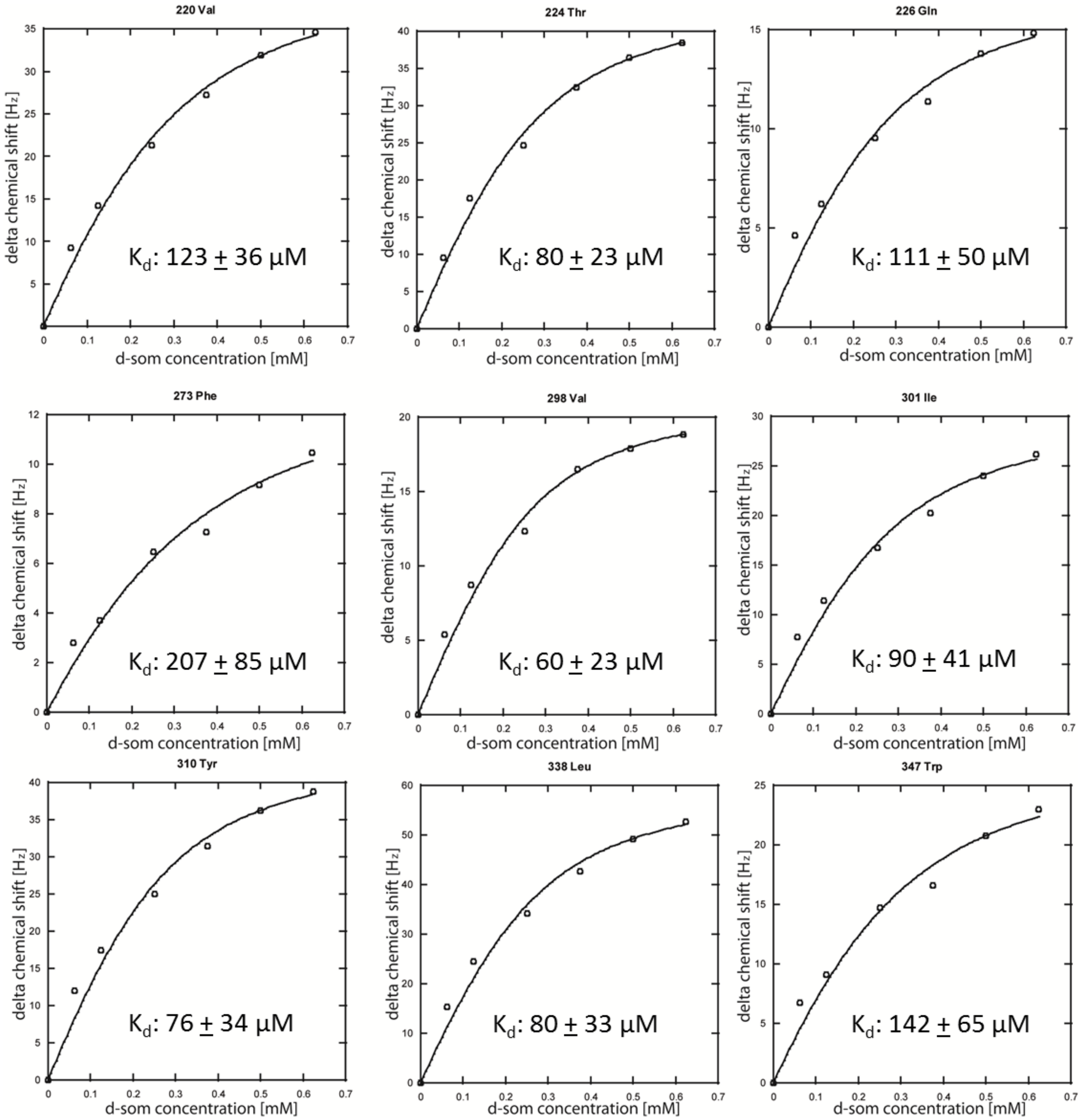

S. IV: $^{15}N,^{1}H$-HSQC spectra of **b'x** + Δ-som (black) overlaid with $^{15}N,^{1}H$-HSQC spectra of **b'x** with the fluorinated and Phe-to-Ala mutant peptides (coloured)

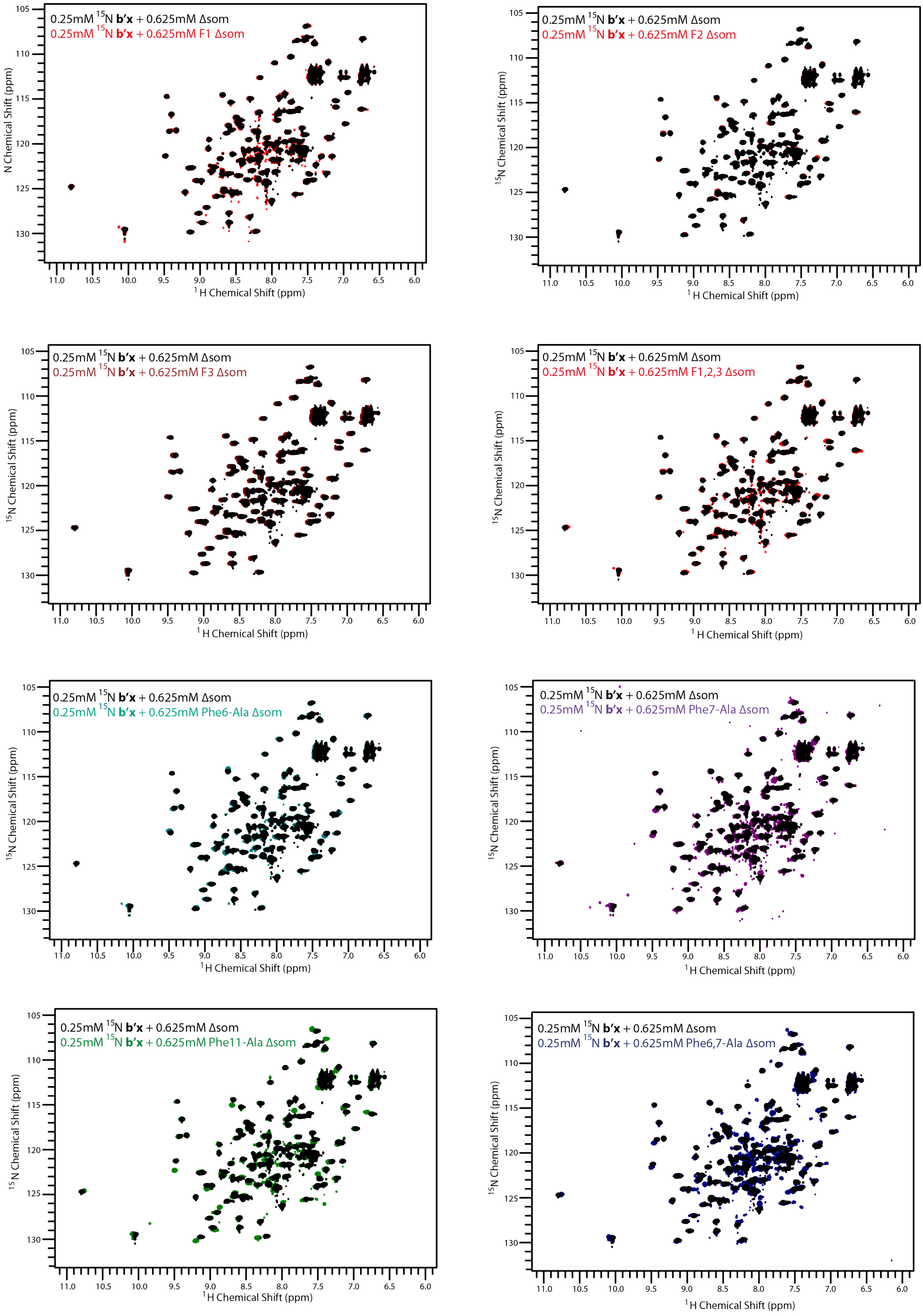

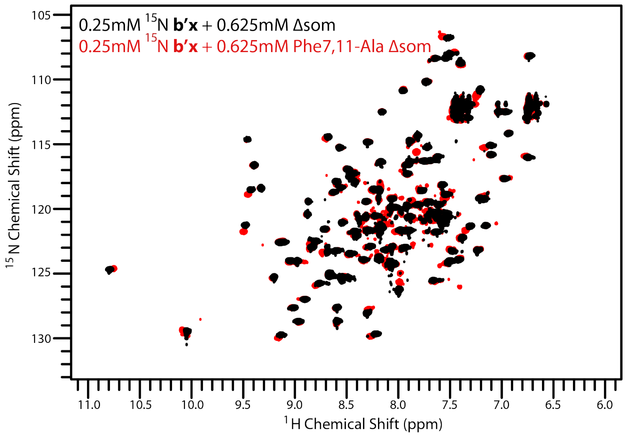

0.25mM ^{15}N **b′x** + 0.625mM Δsom
0.25mM ^{15}N **b′x** + 0.625mM Phe7,11-Ala Δsom
^{15}N Chemical Shift (ppm)
^{1}H Chemical Shift (ppm)

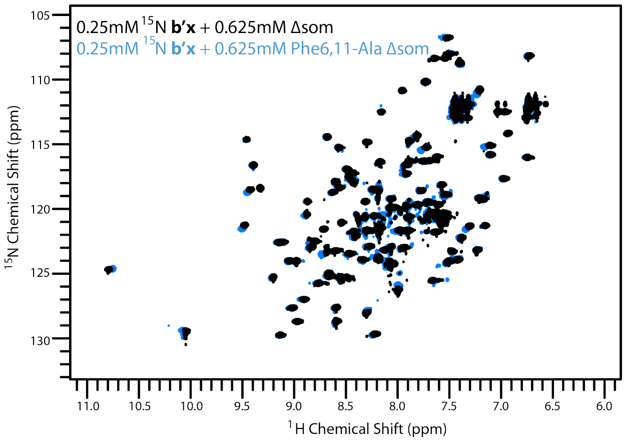

0.25mM ^{15}N **b′x** + 0.625mM Δsom
0.25mM ^{15}N **b′x** + 0.625mM Phe6,11-Ala Δsom
^{15}N Chemical Shift (ppm)
^{1}H Chemical Shift (ppm)

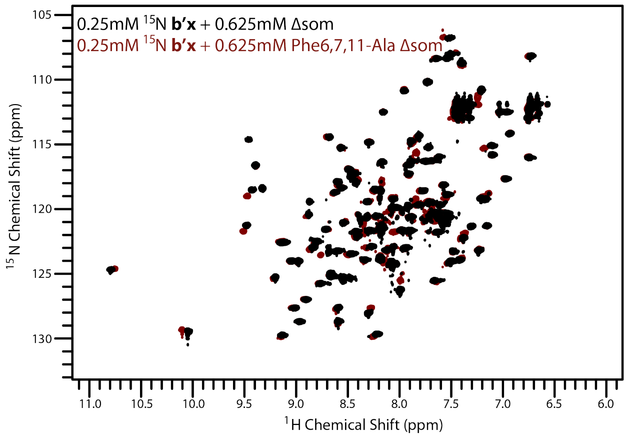

0.25mM ^{15}N **b′x** + 0.625mM Δsom
0.25mM ^{15}N **b′x** + 0.625mM Phe6,7,11-Ala Δsom
^{15}N Chemical Shift (ppm)
^{1}H Chemical Shift (ppm)

S. V: ^{19}F spectra of F1, F2,and F3 Δ-som (0.15mM) with increasing amounts of ^{15}N **b'x**

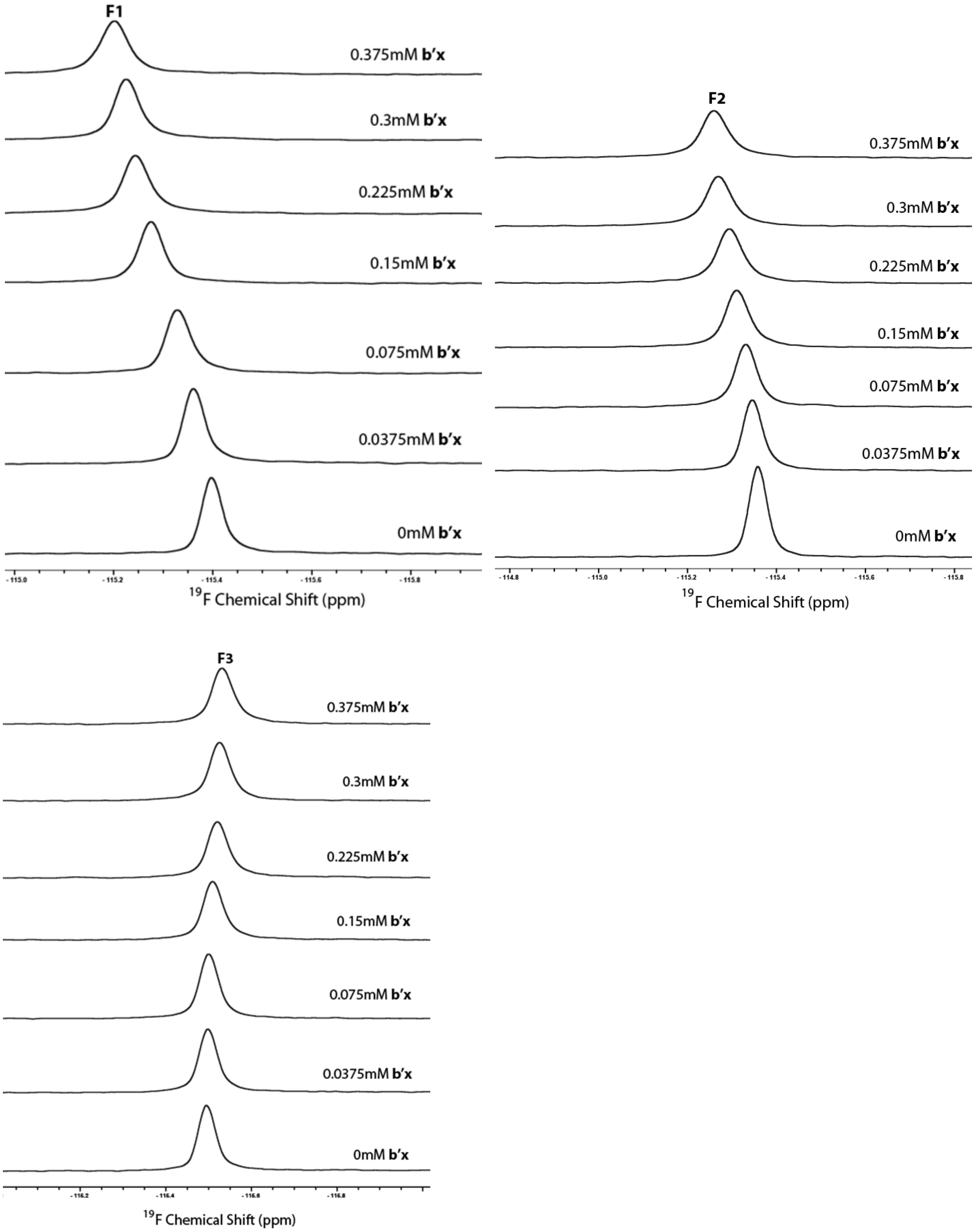

S. VI: ^{15}N,^{1}H-HSQC spectra of ^{15}N **b’x** and ^{15}N **b’x** + 0.625mM Phe6,7,11-Ala Δ-som . Chemical shift perturbation map of 0.25mM **b’x** + 0.625mM Δ-som (blue) and 0.25mM **b’x** + 0.625mM Phe6,7,11-Ala Δ-som (red).

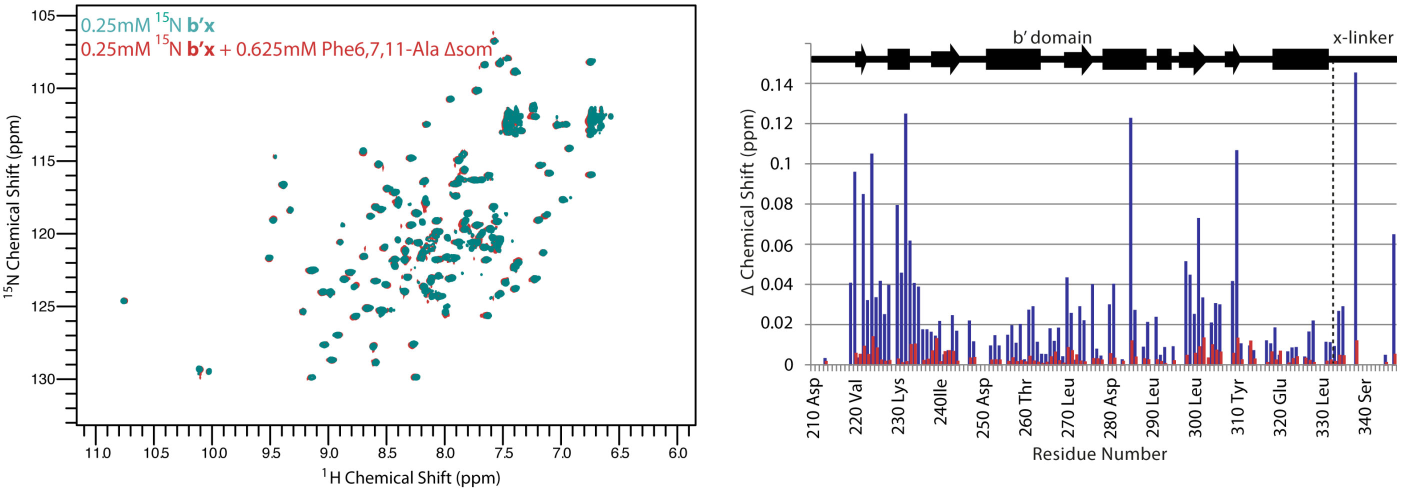

S. VII: ^{15}N,^{1}H-HSQC spectra and chemical shift perturbation map of 0.25mM **b’x** and 0.25mM **b’x** + 0.625mM HSA helix-28.

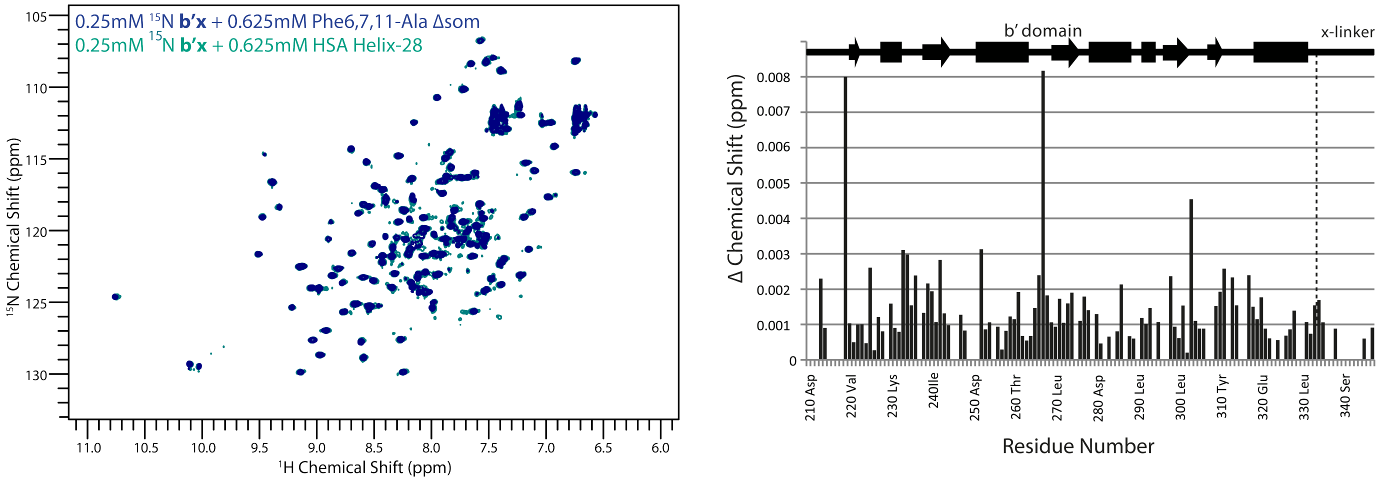

Supplement: Supplementary Information [file srep19518-s1.pdf]
